# Nongenetic Determinants of Risk for Early-Onset Colorectal Cancer

**DOI:** 10.1093/jncics/pkab029

**Published:** 2021-05-20

**Authors:** Alexi N Archambault, Yi Lin, Jihyoun Jeon, Tabitha A Harrison, D Timothy Bishop, Hermann Brenner, Graham Casey, Andrew T Chan, Jenny Chang-Claude, Jane C Figueiredo, Steven Gallinger, Stephen B Gruber, Marc J Gunter, Michael Hoffmeister, Mark A Jenkins, Temitope O Keku, Loïc Le Marchand, Li Li, Victor Moreno, Polly A Newcomb, Rish Pai, Patrick S Parfrey, Gad Rennert, Lori C Sakoda, Robert S Sandler, Martha L Slattery, Mingyang Song, Aung Ko Win, Michael O Woods, Neil Murphy, Peter T Campbell, Yu-Ru Su, Anne Zeleniuch-Jacquotte, Peter S Liang, Mengmeng Du, Li Hsu, Ulrike Peters, Richard B Hayes

**Affiliations:** 1 Division of Epidemiology, Department of Population Health, New York University School of Medicine, New York, NY, USA; 2 Public Health Sciences Division, Fred Hutchinson Cancer Research Center, Seattle, WA, USA; 3 Department of Epidemiology, University of Michigan, Ann Arbor, MI, USA; 4 Leeds Institute of Medical Research at St. James’s, University of Leeds, Leeds, UK; 5 Division of Clinical Epidemiology and Aging Research, German Cancer Research Center (DKFZ), Heidelberg, Germany; 6 Division of Preventive Oncology, German Cancer Research Center (DKFZ) and National Center for Tumor Diseases (NCT), Heidelberg, Germany; 7 German Cancer Consortium (DKTK), German Cancer Research Center (DKFZ), Heidelberg, Germany; 8 Center for Public Health Genomics, University of Virginia, Charlottesville, VA, USA; 9 Division of Gastroenterology, Massachusetts General Hospital and Harvard Medical School, Boston, MA, USA; 10 Channing Division of Network Medicine, Brigham and Women’s Hospital and Harvard Medical School, Boston, MA, USA; 11 Clinical and Translational Epidemiology Unit, Massachusetts General Hospital and Harvard Medical School, Boston, MA, USA; 12 Broad Institute of Harvard and MIT, Cambridge, MA, USA; 13 Department of Epidemiology, Harvard T.H. Chan School of Public Health, Harvard University, Boston, MA, USA; 14 Department of Immunology and Infectious Diseases, Harvard T.H. Chan School of Public Health, Harvard University, Boston, MA, USA; 15 Division of Cancer Epidemiology, German Cancer Research Center (DKFZ), Heidelberg, Germany; 16 University Medical Centre Hamburg-Eppendorf, University Cancer Centre Hamburg (UCCH), Hamburg, Germany; 17 Department of Medicine, Samuel Oschin Comprehensive Cancer Institute, Cedars-Sinai Medical Center, Los Angeles, CA, USA; 18 Department of Preventive Medicine, Keck School of Medicine, University of Southern California, Los Angeles, CA, USA; 19 Lunenfeld Tanenbaum Research Institute, Mount Sinai Hospital, University of Toronto, Toronto, Ontario, Canada; 20 Center for Precision Medicine, City of Hope National Medical Center, Duarte, CA, USA; 21 Nutrition and Metabolism Section, International Agency for Research on Cancer, World Health Organization, Lyon, France; 22 Centre for Epidemiology and Biostatistics, Melbourne School of Population and Global Health, The University of Melbourne, Melbourne, Victoria, Australia; 24 Epidemiology Program, University of Hawaii Cancer Center, Honolulu, HI, USA; 25 Department of Family Medicine, University of Virginia, Charlottesville, VA, USA; 26 Oncology Data Analytics Program, Catalan Institute of Oncology-IDIBELL, L’Hospitalet de Llobregat, Barcelona, Spain; 27 CIBER Epidemiología y Salud Pública (CIBERESP), Madrid, Spain; 28 Department of Clinical Sciences, Faculty of Medicine, University of Barcelona, Barcelona, Spain; 29 ONCOBEL Program, Bellvitge Biomedical Research Institute (IDIBELL), L’Hospitalet de Llobregat, Barcelona, Spain; 30 School of Public Health, University of Washington, Seattle, WA, USA; 31 Department of Laboratory Medicine and Pathology, Mayo Clinic Arizona, Scottsdale, AZ, USA; 32 Memorial University, Faculty of Medicine, Newfoundland, Canada; 33 Department of Community Medicine and Epidemiology, Lady Davis Carmel Medical Center, Haifa, Israel; 34 Ruth and Bruce Rappaport Faculty of Medicine, Technion-Israel Institute of Technology, Haifa, Israel; 35 Clalit National Cancer Control Center, Haifa, Israel; 36 Division of Research, Kaiser Permanente Northern California, Oakland, CA, USA; 37 Center for Gastrointestinal Biology and Disease, University of North Carolina, Chapel Hill, NC, USA; 38 Department of Internal Medicine, University of Utah, Salt Lake City, UT, USA; 39 Department of Nutrition, Harvard T.H. Chan School of Public Health, Harvard University, Boston, MA, USA; 40 Memorial University of Newfoundland, Discipline of Genetics, St John’s, Canada; 41 Section of Nutrition and Metabolism, International Agency for Research on Cancer, Lyon, France; 42 Department of Population Science, American Cancer Society, Atlanta, GA, USA; 43 Biostatistics Unit, Kaiser Permanente Washington Health Research Institute, Seattle, WA, USA; 44 Department of Medicine, New York University School of Medicine, New York, NY, USA; 45 Department of Epidemiology and Biostatistics, Memorial Sloan Kettering Cancer Center, New York, NY, USA; 46 Department of Biostatistics, University of Washington, Seattle, WA, USA; 47 Department of Epidemiology, University of Washington School of Public Health, Seattle, WA, USA

## Abstract

**Background:**

Incidence of early-onset (younger than 50 years of age) colorectal cancer (CRC) is increasing in many countries. Thus, elucidating the role of traditional CRC risk factors in early-onset CRC is a high priority. We sought to determine whether risk factors associated with late-onset CRC were also linked to early-onset CRC and whether association patterns differed by anatomic subsite.

**Methods:**

Using data pooled from 13 population-based studies, we studied 3767 CRC cases and 4049 controls aged younger than 50 years and 23 437 CRC cases and 35 311 controls aged 50 years and older. Using multivariable and multinomial logistic regression, we estimated odds ratios (ORs) and 95% confidence intervals (CIs) to assess the association between risk factors and early-onset CRC and by anatomic subsite.

**Results:**

Early-onset CRC was associated with not regularly using nonsteroidal anti-inflammatory drugs (OR = 1.43, 95% CI = 1.21 to 1.68), greater red meat intake (OR = 1.10, 95% CI = 1.04 to 1.16), lower educational attainment (OR = 1.10, 95% CI = 1.04 to 1.16), alcohol abstinence (OR = 1.23, 95% CI = 1.08 to 1.39), and heavier alcohol use (OR = 1.25, 95% CI = 1.04 to 1.50). No factors exhibited a greater excess in early-onset compared with late-onset CRC. Evaluating risks by anatomic subsite, we found that lower total fiber intake was linked more strongly to rectal (OR = 1.30, 95% CI = 1.14 to 1.48) than colon cancer (OR = 1.14, 95% CI = 1.02 to 1.27; *P* = .04).

**Conclusion:**

In this large study, we identified several nongenetic risk factors associated with early-onset CRC, providing a basis for targeted identification of those most at risk, which is imperative in mitigating the rising burden of this disease.

For the past several decades, early-onset colorectal cancer (CRC; in persons younger than 50 years of age) has been increasing in incidence in many countries (1-10). In the United States, incidence rates of early-onset CRC differ by geographic location and have nearly doubled between 1992 and 2013 (from 8.6 to 13.1 per 100 000 persons) (5), with a preponderance of this increase due to early-onset cancers of the rectum (5,11). The recent rise in early-onset CRC has been observed particularly among individuals born during and after the 1960s in studies from the United States (5,12,13), Canada (3), Australia (1), and Japan (14), suggesting that the differential rates over time are largely attributable to changes in risk factor patterns throughout successive generations.

There is a great need to understand the factors driving the increased incidence of early-onset CRC, because approximately 1 in 10 diagnoses of CRC in the United States occurs in this age group, and these early-onset cancers tend to present with higher pathologic grade and a greater risk of recurrence and metastatic disease (7). Although genetic syndromes (15) and common genetic variants (16) are important in early-onset CRC, the prevalence in young adults of anthropometric, dietary, lifestyle, and pharmacological risk factors for CRC may contribute greatly to the secular trends in early-onset CRC, overall (1,3,5,13) and by anatomic subsite (5,11,13,17‐19). Research in electronic health record databases and small-scale interview-based epidemiologic studies has pointed to potential risk factors for early-onset CRC, including greater consumption of processed meat (20), reduced consumption of vegetables and citrus fruit (20), greater body mass index (BMI) (21‐24), sedentary lifestyle (25), greater alcohol use (20,21,24), smoking (21,22,24), reduced aspirin use (26), and diabetes mellitus (21). However, a comprehensive, large-scale evaluation that compares the magnitude of these risks with those for late-onset CRC (50 years of age and older) and assesses whether the risks for early-onset CRC correlate with specific CRC anatomic subsites has yet to be conducted.

By pooling data from 3 large CRC consortia, we studied whether established anthropometric, dietary, lifestyle, and pharmacological risk factors for late-onset CRC were also linked to early-onset CRC and whether these risks differed from risks for late-onset CRC. Furthermore, we explored whether these risk factors may explain the rising incidence of early-onset CRC by site-specific patterns.

## Methods

### Study Participants

From 3 large consortia—the Colon Cancer Family Registry, the Colorectal Transdisciplinary study, and the Genetics and Epidemiology of Colorectal Cancer Consortium—including 67 168 CRC cases and 710 377 controls, we identified epidemiologic studies that surveyed for detailed CRC risk factors and included a minimum of 20 early-onset CRC cases (younger than 50 years of age at diagnosis). The 13 studies included 3767 CRC cases and 4049 participant controls aged younger than 50 years at diagnosis of the first primary CRC for cases and age at selection for controls (Supplementary Table 1, available online) [for additional study information, see earlier publications (27‐36)]. These same studies also included 23 437 CRC cases and 35 311 controls with a diagnostic or control selection age of 50 years and older (Supplementary Table 2, available online). Cases were confirmed by medical record, pathology report, or death certificate. Controls were identified based on study-specific eligibility and matching criteria, if applicable, which consisted predominantly of age and sex. Participant recruitment across all studies occurred between the 1990s and the early 2010s. Analyses were restricted to participants of genetically defined European descent. All study participants provided written informed consent, and the research was approved by their respective institutional review boards.

### Statistical Analysis

#### Risk Factors and Overall Early-Onset Disease

Risks for colorectal cancer were assessed for 16 self-reported anthropometric, dietary, lifestyle, and pharmacological risk factors. All self-reported variables were ascertained at the reference time for each study, defined as patient selection or blood collection for cohort studies and 1-2 years prior to selection for case-control studies, to ensure exposures were assessed before cancer diagnoses. For studies that assessed height and BMI via direct measurement, variables were captured at the reference time of each respective study. To ensure comparability of variables across studies, all data underwent a multiphase, iterative harmonization process (see the Supplementary Methods, available online) (27,37). Briefly, variables were grouped into a single dataset with universal definitions, standardized coding, and acceptable values. Quality-control checks were implemented, and any values deemed outliers were truncated to a designated range for each respective variable. To address missing data for the examined risk factors, we performed sex- and study-specific mean imputation across the complete consortia dataset (Supplementary Table 3, available online).

Educational attainment was defined as the highest level completed and categorized as the following: less than high school graduate, high school graduate or completed general education development, some college or technical school, and college graduate and higher. Height was represented in increments of 10 cm and captured through either self-report or direct measurement at baseline. BMI, per 5 kg/m^2^, was estimated based on body weight (kg) and height (m^2^) via either self-report or direct measurement at baseline. History of diabetes was characterized as diagnosis of type 2 diabetes at baseline. Smoking was defined using pack-years of smoking among current and former smokers and modeled as study- and sex-specific quartiles. Presence of a sedentary lifestyle was defined as yes (binary) if moderate and/or vigorous physical activity, leisure time, and undifferentiated activities took place less than 1 hour per week. Alcohol intake was categorized according to the grams of alcohol intake per day (14 grams is equivalent to 1 drink): less than 1 g/day (ie, nondrinker), 1-28 g/day, and more than 28 g/day. Aspirin and nonaspirin nonsteroidal anti-inflammatory drug (NSAID) use was defined as yes (binary) if regular use was reported. Dietary factors were captured using food frequency questionnaires or diet histories and included fruit intake (servings/day), vegetable intake (servings/day), red meat intake (servings/day), processed meat intake (servings/day), total calcium intake (mg/day), total folate intake (mcg/day), and total dietary fiber intake (g/day). All dietary variables were modeled as sex- and study-specific quartiles. For all variables, the referent level was the category linked to the lowest risk for CRC based on previously published studies such that the effect estimates for each factor would represent an increase in CRC risk (27,37). Family history of CRC was defined as having 1 or more first-degree relatives with CRC.

We used logistic regression to assess the association between each risk factor and early-onset CRC, adjusting for age, sex, study, family history, and total energy consumption (for dietary factors) (ie, minimally adjusted models). To evaluate the independent effect of these factors on early-onset CRC risk, we used logistic regression incorporating all 16 risk factors, adjusting for age, sex, study, family history, and total energy consumption (ie, multivariable model). We also assessed these relationships for late-onset CRC following the same procedures as for early-onset CRC but additionally accounting for history of screening in the models. Notably, screening for individuals aged 50 years or younger was not standard practice in these regions during the period in which these patients were ascertained, except for possible high-risk families, thus screening history was not accounted for in early-onset models.

Potential heterogeneity across studies was accounted for using random-effects logistic regression; however, results were nearly identical to those from traditional logistic regression models, thus the simpler models were presented here. Statistical assumptions and outliers were evaluated for all models and addressed when necessary. Analyses were completed using the R statistical software program version 3.5.1. All tests were 2-sided, and a *P* value of less than .05 was considered statistically significant.

#### Risk Factors and Disease Site

Because time trend analyses for early-onset CRC suggest that increases in rectal cancer tend to predominate (5,11,13,17), we used multinomial logistic regression to assess the association of risk factors with early-onset rectal cancer and early-onset colon cancer. To test for differences in associations between disease subsites, we applied χ^2^ tests to assess for contrasts in coefficients. Models were adjusted for age, sex, study, family history, and total energy consumption (for dietary factors). Further stratification by anatomic subsite, namely distal colon, proximal colon, and rectum, were also explored for associations with risk factors using a similar approach as described above.

#### Sensitivity Analyses

Sensitivity analyses were performed to evaluate robustness of the results using the mean imputation approach to the presence of missing data. We ran minimally adjusted logistic models for each individual risk factor without imputation (limited to study participants with complete data for that factor); we also ran similar multinomial logistic models to assess these risks by anatomic subsite. In addition, we applied multiple imputation with chained equations (38) to the entire early-onset study group as a second sensitivity analysis.

## Results

### Risk Factors and Overall Early-Onset Disease

Early-onset CRC cases and controls were similar in reference age (45.0 years and 44.7 years, respectively), and men and women were approximately equally distributed across the 2 groups, as expected because of matching on these variables for many of the included studies (Table 1). Cases aged younger than 50 years were predominantly located in the rectum (39.8%), followed by the distal colon (32.3%) and the proximal colon (27.9%).

**Table 1.** Baseline participant characteristics of participants aged younger than 50 years^a^

Characteristic	Cases	Controls
Total No.	3767	4049
Age, mean (SD)	45.01 (7.85)	44.73 (5.47)
Age, No. (%), y		
<30	123 (3.3)	130 (3.2)
30-40	2842 (75.4)	3043 (75.2)
>40	802 (21.3)	876 (21.6)
Sex, No. (%)		
Female	1948 (51.7)	2089 (51.6)
Male	1819 (48.3)	1960 (48.4)
Disease site, No. (%)		
Proximal colon	966 (27.9)	—
Distal colon	1117 (32.3)	—
Rectum	1379 (39.8)	—
Education, highest level completed, No. (%)		
< High school graduate	490 (14.0)	622 (16.0)
High school graduate or completed GED	766 (21.9)	539 (13.9)
Some college or technical school	1060 (30.3)	1192 (30.7)
≥ College graduate	1185 (33.8)	1531 (39.4)
Family history, No. (%)		
No	2597 (77.3)	2391 (80.9)
Yes	763 (22.7)	566 (19.1)
Height, per 10 cm, mean (SD)	17.13 (1.01)	17.08 (0.95)
BMI, per 5 kg/m^2^, mean (SD)	5.45 (1.12)	5.39 (1.03)
Red meat, No. (%), servings/d		
Quartile 1^b^	863 (24.6)	1060 (26.8)
Quartile 2^b^	758 (21.6)	1230 (31.1)
Quartile 3^b^	875 (25.0)	1009 (25.5)
Quartile 4^b^	1006 (28.7)	652 (16.5)
Processed meat, No. (%), servings/d		
Quartile 1^b^	243 (12.0)	388 (12.5)
Quartile 2^b^	604 (29.9)	1073 (34.7)
Quartile 3^b^	767 (38.0)	1359 (43.9)
Quartile 4^b^	406 (20.1)	274 (8.9)
Fruit, No. (%), servings/d		
Quartile 1^b^	1388 (39.6)	1471 (37.4)
Quartile 2^b^	833 (23.8)	982 (25.0)
Quartile 3^b^	723 (20.6)	772 (19.6)
Quartile 4^b^	560 (16.0)	707 (18.0)
Vegetable, No. (%), servings/d		
Quartile 1^b^	861 (24.3)	1236 (31.3)
Quartile 2^b^	1308 (36.9)	1130 (28.6)
Quartile 3^b^	909 (25.7)	906 (22.9)
Quartile 4^b^	463 (13.1)	677 (17.1)
Total fiber, No. (%), g/d		
Quartile 1^b^	379 (27.6)	235 (26.9)
Quartile 2^b^	337 (24.5)	211 (24.2)
Quartile 3^b^	306 (22.3)	206 (23.6)
Quartile 4^b^	353 (25.7)	221 (25.3)
Total calcium intake, No. (%), mg/d		
Quartile 1^b^	290 (9.2)	193 (5.2)
Quartile 2^b^	1816 (57.3)	2442 (65.3)
Quartile 3^b^	802 (25.3)	873 (23.4)
Quartile 4^b^	261 (8.2)	229 (6.1)
Total folate intake, No. (%), mcg/d		
Quartile 1^b^	497 (19.9)	238 (7.4)
Quartile 2^b^	1040 (41.7)	1999 (62.3)
Quartile 3^b^	691 (27.7)	795 (24.8)
Quartile 4^b^	268 (10.7)	178 (5.5)
Sedentary lifestyle, No. (%)		
No	716 (79.3)	1769 (82.4)
Yes	187 (20.7)	377 (17.6)
Pack-years of smoking, No. (%)		
Never smoker	1848 (56.2)	2240 (62.4)
Quartile 1^b^	457 (13.9)	451 (12.6)
Quartile 2^b^	440 (13.4)	387 (10.8)
Quartile 3^b^	384 (11.7)	355 (9.9)
Quartile 4^b^	162 (4.9)	155 (4.3)
Alcohol use, No. (%)		
0 g/day	1435 (43.1)	1123 (28.4)
1-28 g/day	1472 (44.2)	2284 (57.8)
>28 g/day	424 (12.7)	547 (13.8)
Aspirin use, No. (%)		
No	3253 (91.7)	3647 (92.0)
Yes	296 (8.3)	315 (8.0)
NSAID use, No. (%)		
No	3152 (89.4)	3262 (82.6)
Yes	375 (10.6)	689 (17.4)
History of diabetes, No. (%)		
No	3425 (95.3)	3823 (97.3)
Yes	168 (4.7)	108 (2.7)

a Age defined as the age of diagnosis of the first primary CRC for cases and as the age at selection for controls. — = participants do not have data for “Disease site”; BMI = body mass index; CRC = colorectal cancer; GED = general educational development.

b Study and sex-specific quartiles.

**Figure 1.**
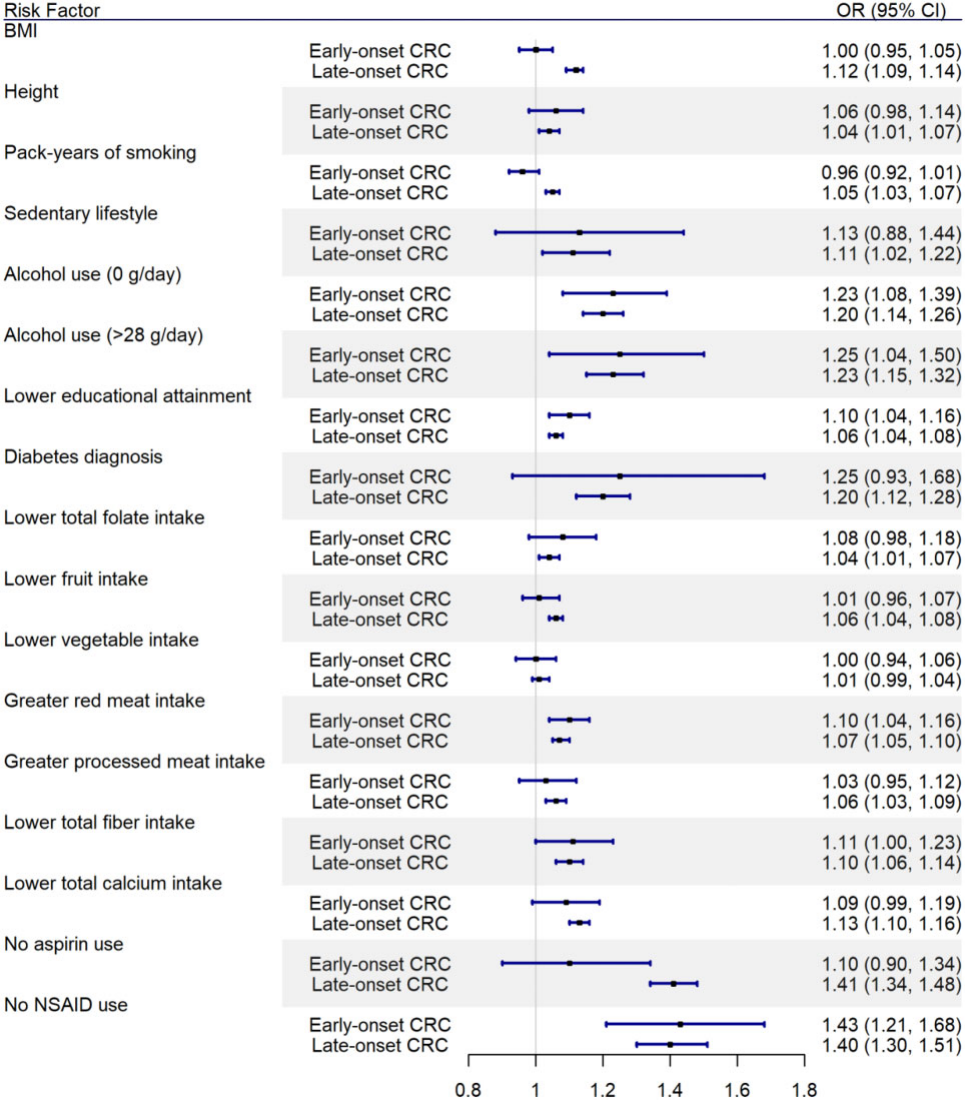
Risk estimates for early-onset vs late-onset colorectal cancer (CRC) associated with anthropometric, dietary, lifestyle, and pharmacological risk factors. Data presented from multivariable models, which were adjusted for age, sex, study, family history, and total energy consumption; the late-onset model was additionally adjusted for history of screening. Dietary variables were harmonized across studies by sex- and study-specific quartiles, and assigned values 0, 1, 2, and 3 in the order of increasing risk. These variables were treated as continuous variables in the analysis. Error bars indicate 95% confidence intervals. BMI = body mass index; CI = confidence interval; NSAID = nonsteroidal anti-inflammatory drug; OR = odds ratio.

We found that early-onset CRC was associated with several factors previously linked to CRC overall, in minimally adjusted (Table 2) and multivariable models (Table 2 and Figure 1). In multivariable models, early-onset CRC was associated with not regularly using NSAIDs (OR = 1.43, 95% CI = 1.21 to 1.68), greater red meat intake (OR = 1.10, 95% CI = 1.04 to 1.16), lower educational attainment (OR = 1.10, 95% CI = 1.04 to 1.16), and alcohol abstinence (OR = 1.23, 95% CI = 1.08 to 1.39) and heavier alcohol use (>28 g/day of alcohol; OR = 1.25, 95% CI = 1.04 to 1.50). Several other CRC risk factors trended toward an association with early-onset CRC in multivariable models, including history of diabetes and lower folate, dietary fiber, and calcium intake. Comparing risk factors between early and late-onset CRC in multivariable models, we found that no factors appreciably exhibited a greater excess in effect size for early-onset compared with late-onset cancer (Supplementary Table 4, available online; Figure 1). However, several risk factors were suggestive of carrying greater risk for late-onset compared with early-onset CRC, including BMI, smoking, and no use of aspirin. To account for possible confounding by indication due to inflammatory bowel disease in the relationship between NSAID use and risk for early-onset CRC, a sensitivity analysis restricted to individuals without a confirmed inflammatory bowel disease diagnosis (n = 4220) was carried out, and results remained unchanged (Supplementary Table 5, available online).

**Table 2.** Risk estimates for early-onset colorectal cancer associated with anthropometric, dietary, lifestyle, and pharmacological risk factors

Lifestyle and environmental risk factor^a^	Minimally adjusted models^b^		Multivariable model^c^	
	OR (95% CI)	*P*	OR (95% CI)	*P*
Anthropometric				
BMI, per 5 kg/m^2^	1.03 (0.98 to 1.08)	.28	1.00 (0.95 to 1.05)	.95
Height, per 10 cm	1.03 (0.96 to 1.11)	.43	1.06 (0.98 to 1.14)	.16
Lifestyle				
Pack-years of smoking	0.99 (0.95 to 1.04)	.69	0.96 (0.92 to 1.01)	.12
Sedentary lifestyle	1.13 (0.90 to 1.42)	.31	1.13 (0.88 to 1.44)	.34
Alcohol use, 0 g/d	1.28 (1.13 to 1.45)	<.001	1.23 (1.08 to 1.39)	.001
Alcohol use, >28 g/d	1.31 (1.11 to 1.55)	.002	1.25 (1.04 to 1.50)	.02
Lower educational attainment, highest level completed	1.12 (1.07 to 1.18)	<.001	1.10 (1.04 to 1.16)	<.001
History of diabetes	1.24 (0.94 to 1.64)	.12	1.25 (0.93 to 1.68)	.14
Dietary				
Lower total folate intake, mcg/d^d^	1.16 (1.08 to 1.26)	<.001	1.08 (0.98 to 1.18)	.11
Lower fruit intake, servings/d^d^	1.07 (1.02 to 1.12)	.008	1.01 (0.96 to 1.07)	.69
Lower vegetable intake, servings/d^d^	1.05 (0.99 to 1.10)	.08	1.00 (0.94 to 1.06)	.98
Greater red meat intake, servings/d^d^	1.12 (1.07 to 1.18)	<.001	1.10 (1.04 to 1.16)	<.001
Greater processed meat intake, servings/d^d^	1.08 (1.00 to 1.16)	.06	1.03 (0.95 to 1.12)	.43
Lower total fiber intake, g/d^d^	1.19 (1.08 to 1.31)	<.001	1.11 (1.00 to 1.23)	.06
Lower total calcium intake, mg/d^d^	1.17 (1.08 to 1.28)	<.001	1.09 (0.99 to 1.19)	.08
Pharmacological				
No aspirin use	1.07 (0.88 to 1.29)	.51	1.10 (0.90 to 1.34)	.36
No NSAID use	1.43 (1.22 to 1.68)	<.001	1.43 (1.21 to 1.68)	<.001

a The referent category for each categorical factor was defined as the following: presence of a sedentary lifestyle (no), alcohol intake (1-28 g/day), educational attainment (≥ college graduate), history of diabetes (no), aspirin use (yes), and NSAID use (yes). BMI = body mass index; CI = confidence interval; NSAID = nonsteroidal anti-inflammatory drug; OR = odds ratio.

b Logistic regression models include individual nongenetic factors and were adjusted for age, sex, study, family history, and total energy consumption (for dietary factors).

c Logistic regression model includes all nongenetic factors and was adjusted for age, sex, study, family history, and total energy consumption.

d Dietary variables were harmonized across studies by sex- and study-specific quartiles and assigned values 0, 1, 2, and 3 in the order of increasing risk. These variables were treated as continuous variables in the analysis.

### Risk Factors and Disease Site

Evaluating risks for early-onset CRC by cancer subsite (Table 3), we found that not regularly using NSAIDs, greater red meat intake, lower dietary fiber intake, lower folate intake, lower calcium intake, alcohol abstinence and heavier alcohol use (>28 g/day of alcohol), and lower educational attainment were all linked to greater risk for both rectal and colon early-onset disease. Further contrasting these associations between subsite, lower total dietary fiber intake was associated more strongly with rectal (OR = 1.30, 95% CI = 1.14 to 1.48) than colon cancer (OR = 1.14, 95% CI = 1.02 to 1.27; *P* = .04). Several other risk factors tended toward a greater risk for rectal cancer, including no regular use of NSAIDs and lower folate intake. After further stratification across anatomic subsites (Supplementary Table 6, available online), lower total fiber intake was more closely associated with cancers of the proximal colon (OR = 1.24, 95% CI = 1.08 to 1.43) compared with those of the distal region (OR = 1.06, 95% CI = 0.94 to 1.21; *P* = .05).

**Table 3.** Association between anthropometric, dietary, lifestyle, and pharmacological risk factors and early-onset colorectal cancer risk, stratified by anatomic subsite

Lifestyle and environmental risk factor^a^	Colon cancer^b^		Rectal cancer^b^		Colon vs rectum^c^
	OR (95% CI)	*P*	OR (95% CI)	*P*	*P*
Anthropometric					
BMI, per 5 kg/m^2^	1.05 (0.99 to 1.10)	.09	0.99 (0.93 to 1.06)	.84	.11
Height, per 10 cm	1.03 (0.94 to 1.12)	.55	1.03 (0.93 to 1.13)	.57	.96
Lifestyle					
Pack-years of smoking	0.99 (0.94 to 1.04)	.69	0.99 (0.94 to 1.05)	.73	.99
Sedentary lifestyle	1.15 (0.88 to 1.51)	.30	1.09 (0.78 to 1.53)	.63	.77
Alcohol use, 0 g/d	1.28 (1.12 to 1.47)	<.001	1.30 (1.11 to 1.53)	.001	.86
Alcohol use, >28 g/d	1.29 (1.06 to 1.57)	.01	1.34 (1.08 to 1.67)	.009	.75
Lower educational attainment, highest level completed	1.12 (1.05 to 1.18)	<.001	1.13 (1.06 to 1.21)	<.001	.68
History of diabetes	1.20 (0.88 to 1.63)	.25	1.28 (0.90 to 1.81)	.16	.70
Dietary					
Lower total folate intake, mcg/d^d^	1.14 (1.04 to 1.24)	.003	1.24 (1.11 to 1.37)	<.001	.12
Lower fruit intake, servings/d^d^	1.05 (0.99 to 1.10)	.09	1.10 (1.03 to 1.17)	.004	.16
Lower vegetable intake, servings/d^d^	1.03 (0.97 to 1.10)	.28	1.08 (1.01 to 1.16)	.03	.24
Greater red meat intake, servings/d^d^	1.12 (1.06 to 1.18)	<.001	1.12 (1.05 to 1.19)	.001	.99
Greater processed meat intake (servings/day)^d^	1.06 (0.97 to 1.16)	.18	1.09 (0.98 to 1.21)	.11	.65
Lower total fiber intake, g/d^d^	1.14 (1.02 to 1.27)	.02	1.30 (1.14 to 1.48)	<.001	.04
Lower total calcium intake, mg/d^d^	1.15 (1.05 to 1.26)	.003	1.24 (1.11 to 1.39)	<.001	.18
Pharmacological					
No aspirin use	1.15 (0.92 to 1.42)	.21	1.04 (0.81 to 1.34)	.75	.47
No NSAID use	1.33 (1.12 to 1.60)	.002	1.66 (1.31 to 2.09)	<.001	.08

a The referent category for each categorical factor was defined as the following: presence of a sedentary lifestyle (no), alcohol intake (1-28 g/d), educational attainment (≥ college graduate), history of diabetes (no), aspirin use (yes), and NSAID use (yes). BMI = body mass index; CI = confidence interval; NSAID = nonsteroidal anti-inflammatory drug; OR = odds ratio.

b Multinomial logistic regression models include individual nongenetic factors and were adjusted for age, sex, study, family history, and total energy consumption (for dietary factors).

c χ^2^ test for contrasts in multinomial models.

d Dietary variables were harmonized across studies by sex- and study-specific quartiles and assigned values 0, 1, 2, and 3 in the order of increasing risk. These variables were treated as continuous variables in the analysis.

### Sensitivity Analyses

By comparing risk estimates from minimally adjusted logistic models produced using data with mean imputation (Table 2) with those generated using multiple imputation or the reduced complete case data (Supplementary Table 7, available online), we found the effect sizes were almost identical in magnitude. Similarly, effect estimates from minimally adjusted multinomial logistic models produced using data with mean imputation (Table 3) and those generated using complete case data (Supplementary Table 8, available online) were almost identical in magnitude.

## Discussion

Our study, including 3767 early-onset CRC and 4049 controls, demonstrated that several nongenetic factors known to be involved in late-onset CRC (27,37) are also relevant for early-onset disease. In particular, not regularly using NSAIDs, greater red meat intake, alcohol abstinence and heavier alcohol use, and lower educational attainment were statistically significantly associated with early-onset CRC. Notably, this study is novel in that it statistically examined how associations between risk factors and early-onset CRC differ by subsite. In doing so, we provide the first evidence that no use of NSAIDs, lower intake of dietary fiber, and lower intake of folate may be more strongly associated with early-onset cancers of the rectum, compared with those of the colon.

Pharmacological, dietary, lifestyle, and anthropometric-related risk factors for CRC have been clearly established for late-onset disease (27,37); however, research on these factors in early-onset CRC is less developed, relying often on smaller studies and examination of a limited number of risk factors. Evidence on pharmacological factors and early-onset CRC is limited, although lower aspirin use was related to greater risk of CRC in 1 study (26). As diets have shifted considerably over the past several decades, several researchers hypothesize that dietary factors are largely driving the higher rates of CRC in younger individuals. Reduced intake of folate (20), calcium (20), citrus fruits (20), and greater processed meat (20) has demonstrated a positive association in some studies with greater risk of early-onset CRC. Certain lifestyle factors have also been suggested to increase one’s risk for early-onset CRC, including smoking (21,22,24,39,40), a sedentary lifestyle (25), abstinence or heavy alcohol use (20,24,39), and a history of diabetes (22,40). Lastly, associations between greater BMI and risk of early-onset CRC have been inconsistently shown (22‐24,26,39,40). Our larger, comprehensive study generally tended to replicate previous reports, although some differences were noteworthy. In particular, neither BMI nor smoking were risk factors in our early-onset series, in contrast to the late-onset group.

The recent rise internationally in early-onset CRC incidence is related, to a substantial degree, to increases in rectal cancer (5,11,13,17). Although prior work has shown that select dietary factors, including calcium and fiber intake (41), and aspirin (18,41) tend to exert greater risk over all ages combined for rectal cancer compared with colon cancer (18,19,42), studies have yet to reveal such differences for early-onset disease. However, previous studies were small or included a broader definition of early-onset CRC up to 60 years of age (18,41). Thus, our study is the first to identify statistically significant differences in early-onset CRC by disease subsite, particularly for dietary fiber and possibly for no use of NSAIDs and lower intake of folate.

Whereas early-onset CRC has been characterized by a greater preponderance of rectal cancer, temporal increases associated with birth cohort effects have also been noted (1,3,5,13), thus suggesting that risk factors strongly linked with rectal cancer and increasing in prevalence may explain the increasing rates of early-onset disease. Major shifts in dietary consumption in the past decades among younger generations are well established for the United States (43) and internationally (44) characterized typically by decreases in consumption of fruits, non-potato vegetables, and calcium-rich dairy sources, coupled with an increase in processed foods (eg, meats, pizza, macaroni and cheese) and soft beverages. Concurrent with changes in foods consumed, nutrient intakes of fiber, folate, and calcium are lower than dietary recommendations among US adolescents (43), although current folate intake likely has increased recently because of folic acid fortification of all enriched cereal-grain products by the Food and Drug Administration beginning in 1998 (45). Furthermore, adolescent use of NSAIDs has decreased over recent generations (46). Consistent with these trends, we identified several factors, including no use of NSAIDs and lower intake of several dietary factors, that tended toward greater association with rectal compared with colon cancer. These findings may provide the first clues that generational changes in risk-related exposures may contribute to the increases observed internationally in early-onset CRC.

Our study is among the first to comprehensively assess the relationship of well-established CRC risk factors in the development of early-onset CRC. We leveraged multiple studies from heterogeneous populations, and we included rigorous harmonization across these studies of risk factors and disease phenotypes (27,37). Despite these strengths, this research also has limitations. Anthropometric, dietary, lifestyle, and pharmacological risk factors were self-reported, which may result in misclassification, although prior work has shown that self-reported lifestyle and diet are relatively accurate (47,48). Second, sex- and study-specific mean imputation for addressing missing data reduced the variance of distributions, potentially resulting in biased estimates; however, sensitivity analyses using complete case data or multiple imputation did not produce substantial differences. As with all studies using pooled data, heterogeneity stemming from study design is a potential concern; this points to the need for additional large cohort studies to assess these relationships. For case-control studies, risk factors were assessed after cancer diagnosis, which therefore makes their data susceptible to recall bias. Nevertheless, relative risks for each known risk factor (Table 2) were relatively comparable to those previously reported throughout the literature. Further, measurement error in the dietary assessment of energy may have had a noteworthy impact on the presence of residual confounding for dietary factors. Prior weight loss due to CRC manifestation may have biased BMI ascertainment and likely may explain our null findings for BMI risk; additional analyses using prospective cohorts or Mendelian randomization methods are warranted to elucidate this association. Additionally, we note that the observed differentials in risk by disease subsite may be influenced by multiple testing and require further independent validation. Lastly, only individuals of European ancestry were included, thus limiting the generalizability of the findings. Associations may differ across racial and ethnic populations, emphasizing the need for racially and ethnically diverse cohorts, particularly as early-onset CRC occurs more commonly among Black, Asian, Pacific Islander, and Hispanic communities (49‐51).

In summary, we found that a subset of established nongenetic risk factors for late-onset CRC were additionally related to early-onset CRC. Our research also provided the first evidence linking CRC risk factors to early-onset anatomic subsite patterns, specifically for lower intake of dietary fiber. These results present key insights concerning risk factors that contribute to CRC manifestation in younger individuals, providing a basis for identification of those most at risk, which is imperative in mitigating the rising burden of this disease.

## Funding

This work was funded by the National Cancer Institute under R03-CA215775-02, awarded to Dr Richard Hayes, and through the Genetics and Epidemiology of Colorectal Cancer Consortium (GECCO) funded by the National Cancer Institute, National Institutes of Health, US Department of Health and Human Services (U01 CA164930, R01 CA201407), awarded to Dr Ulrike Peters. This research was funded in part through the NIH/NCI Cancer Center Support Grant P30 CA015704 and training grant T32HS026120, from the Agency for Healthcare Research and Quality. The Colon Cancer Family Registry (CCFR, www.coloncfr.org) is supported in part by funding from the National Cancer Institute (NCI), National Institutes of Health (NIH) (award U01 CA167551). The CCFR Set-1 (Illumina 1 M/1M-Duo) and Set-2 (Illumina Omni1-Quad) scans were supported by NIH awards U01 CA122839 and R01 CA143247 (to GC). The CCFR Set-3 (Affymetrix Axiom CORECT Set array) was supported by NIH award U19 CA148107 and R01 CA81488 (to SBG). The CCFR Set-4 (Illumina OncoArray 600 K SNP array) was supported by NIH award U19 CA148107 (to SBG) and by the Center for Inherited Disease Research (CIDR), which is funded by the NIH to the Johns Hopkins University, contract number HHSN268201200008I. CRCGEN: Colorectal Cancer Genetics & Genomics, Spanish study was supported by Instituto de Salud Carlos III, co-funded by FEDER funds -a way to build Europe- (grants PI14-613 and PI09-1286), Agency for Management of University and Research Grants (AGAUR) of the Catalan Government (grant 2017SGR723), and Junta de Castilla y León (grant LE22A10-2). Sample collection of this work was supported by the Xarxa de Bancs de Tumors de Catalunya sponsored by Pla Director d’Oncología de Catalunya (XBTC), Plataforma Biobancos PT13/0010/0013 and ICOBIOBANC, sponsored by the Catalan Institute of Oncology. DACHS: This work was supported by the German Research Council (BR 1704/6-1, BR 1704/6-3, BR 1704/6-4, CH 117/1-1, HO 5117/2-1, HE 5998/2-1, KL 2354/3-1, RO 2270/8-1 and BR 1704/17-1), the Interdisciplinary Research Program of the National Center for Tumor Diseases (NCT), Germany, and the German Federal Ministry of Education and Research (01KH0404, 01ER0814, 01ER0815, 01ER1505A and 01ER1505B). DALS: National Institutes of Health (R01 CA48998 to M. L. Slattery). EPIC: The coordination of EPIC is financially supported by the European Commission (DGSANCO) and the International Agency for Research on Cancer. The national cohorts are supported by Danish Cancer Society (Denmark); Ligue Contre le Cancer, Institut Gustave Roussy, Mutuelle Générale de l’Education Nationale, Institut National de la Santé et de la Recherche Médicale (INSERM) (France); German Cancer Aid, German Cancer Research Center (DKFZ), Federal Ministry of Education and Research (BMBF), Deutsche Krebshilfe, Deutsches Krebsforschungszentrum and Federal Ministry of Education and Research (Germany); the Hellenic Health Foundation (Greece); Associazione Italiana per la Ricerca sul Cancro-AIRCItaly and National Research Council (Italy); Dutch Ministry of Public Health, Welfare and Sports (VWS), Netherlands Cancer Registry (NKR), LK Research Funds, Dutch Prevention Funds, Dutch ZON (Zorg Onderzoek Nederland), World Cancer Research Fund (WCRF), Statistics Netherlands (The Netherlands); ERC-2009-AdG 232997 and Nordforsk, Nordic Centre of Excellence programme on Food, Nutrition and Health (Norway); Health Research Fund (FIS), PI13/00061 to Granada, PI13/01162 to EPIC-Murcia, Regional Governments of Andalucía, Asturias, Basque Country, Murcia and Navarra, ISCIII RETIC (RD06/0020) (Spain); Swedish Cancer Society, Swedish Research Council and County Councils of Skåne and Västerbotten (Sweden); Cancer Research UK (14136 to EPIC-Norfolk; C570/A16491 and C8221/A19170 to EPIC-Oxford), Medical Research Council (1000143 to EPIC-Norfolk, MR/M012190/1 to EPIC-Oxford) (United Kingdom). Kentucky: This work was supported by the following grant support: Clinical Investigator Award from Damon Runyon Cancer Research Foundation (CI-8); NCI R01CA136726. LCCS: The Leeds Colorectal Cancer Study was funded by the Food Standards Agency and Cancer Research UK Programme Award (C588/A19167). MECC: This work was supported by the National Institutes of Health, U.S. Department of Health and Human Services (R01 CA81488 to SBG and GR). NCCCS I & II: We acknowledge funding support for this project from the National Institutes of Health, R01 CA66635 and P30 DK034987. NFCCR: This work was supported by an Interdisciplinary Health Research Team award from the Canadian Institutes of Health Research (CRT 43821); the National Institutes of Health, U.S. Department of Health and Human Services (U01 CA74783); and National Cancer Institute of Canada grants (18223 and 18226). The authors wish to acknowledge the contribution of Alexandre Belisle and the genotyping team of the McGill University and Génome Québec Innovation Centre, Montréal, Canada, for genotyping the Sequenom panel in the NFCCR samples. Funding was provided to Michael O. Woods by the Canadian Cancer Society Research Institute. Harvard cohort (NHS): NHS is supported by the National Institutes of Health (R01 CA137178, P01 CA087969, UM1 CA186107, R01 CA151993, R35 CA197735, K07CA190673, and P50 CA127003). OFCCR: The Ontario Familial Colorectal Cancer Registry was supported in part by the National Cancer Institute (NCI) of the National Institutes of Health (NIH) under award U01 CA167551 and award U01/U24 CA074783 (to SG). Additional funding for the OFCCR and ARCTIC testing and genetic analysis was through and a Canadian Cancer Society CaRE (Cancer Risk Evaluation) program grant and Ontario Research Fund award GL201-043 (to BWZ), through the Canadian Institutes of Health Research award 112746 (to TJH), and through generous support from the Ontario Ministry of Research and Innovation. SCCFR: The Seattle Colon Cancer Family Registry was supported in part by the National Cancer Institute (NCI) of the National Institutes of Health (NIH) under awards U01 CA167551, U01 CA074794 (to JDP), and awards U24 CA074794 and R01 CA076366 (to PAN). UK Biobank: This research has been conducted using the UK Biobank Resource under Application Number 8614.

## Notes

**The role of the funders**: The funders had no role in the design of the study, the writing of the manuscript, the decision to submit the manuscript for publication, and the collection, analysis, and interpretation of the data.

**Disclosures**: The authors have no conflicts of interest to report and assume full responsibility for all aspects of this study.

**Author contributions**: ANA: Conceptualization, Formal Analysis, Investigation, Methodology, Writing—original draft, Writing—review & editing; YL: Data curation, Formal Analysis, Writing—review & editing; JJ: Methodology, Writing—review & editing; TAH: Writing—review & editing; DTB: Writing—review & editing; HB: Writing—review & editing; GC: Writing—review & editing; ATC: Writing—review & editing; JC: Writing—review & editing; JCF: Writing—review & editing; SG: Writing—review & editing; SBG: Writing—review & editing; MJG: Writing—review & editing; MH: Writing—review & editing; MAJ: Writing—review & editing; TOK: Writing—review & editing; LM: Writing—review & editing; LL: Writing—review & editing; VM: Writing—review & editing; PAN: Writing—review & editing; RP: Writing—review & editing; PSP: Writing—review & editing; GR: Writing—review & editing; LCS: Writing—review & editing; RSS: Writing—review & editing; MLS: Writing—review & editing; MS: Writing—review & editing; AKW: Writing—review & editing; MOW: Writing—review & editing; NM: Writing—review & editing; PTC: Writing—review & editing; YS: Writing—review & editing; AZ: Methodology, Writing—review & editing; PSL: Methodology, Writing—review & editing; MD: Writing—review & editing; LH: Conceptualization, Formal Analysis, Investigation, Methodology, Supervision, Writing—review & editing; UP: Conceptualization, Investigation, Funding acquisition, Methodology, Supervision, Writing—review & editing; RBH: Conceptualization, Formal Analysis, Investigation, Funding acquisition, Methodology, Supervision, Writing—original draft, Writing—review & editing.

**Acknowledgements**: Participating studies would like to acknowledge the following contributors: DACHS: We thank all participants and cooperating clinicians, and Ute Handte-Daub and Utz Benscheid for excellent technical assistance. EPIC: Where authors are identified as personnel of the International Agency for Research on Cancer/World Health Organization, the authors alone are responsible for the views expressed in this article, and they do not necessarily represent the decisions, policy, or views of the International Agency for Research on Cancer/World Health Organization. Kentucky: We would like to acknowledge the staff at the Kentucky Cancer Registry. LCCS: We acknowledge the contributions of Jennifer Barrett, Robin Waxman, Gillian Smith, and Emma Northwood in conducting this study. NCCCS I & II: We would like to thank the study participants and the NC Colorectal Cancer Study staff. Harvard cohort (NHS): The study protocol was approved by the institutional review boards of the Brigham and Women’s Hospital and Harvard T.H. Chan School of Public Health, and those of participating registries as required. We would like to thank the participants and staff of the HPFS, NHS, and PHS for their valuable contributions as well as the following state cancer registries for their help: AL, AZ, AR, CA, CO, CT, DE, FL, GA, ID, IL, IN, IA, KY, LA, ME, MD, MA, MI, NE, NH, NJ, NY, NC, ND, OH, OK, OR, PA, RI, SC, TN, TX, VA, WA, WY. The authors assume full responsibility for analyses and interpretation of these data. SCCFR: The authors would like to thank the study participants and staff of the Hormones and Colon Cancer and Seattle Cancer Family Registry studies (CORE Studies).

**Disclaimers:** The content is solely the responsibility of the authors and does not necessarily represent the official views of the Agency for Healthcare Research and Quality. The content of this manuscript does not necessarily reflect the views or policies of the NCI, NIH, or any of the collaborating centers in the Colon Cancer Family Registry (CCFR), nor does mention of trade names, commercial products, or organizations imply endorsement by the US government, any cancer registry, or the CCFR.

## Data Availability

The data underlying this article were accessed from the Fred Hutchinson Cancer Center (https://www.fredhutch.org/en/research/divisions/public-health-sciences-division/research/cancer-prevention/genetics-epidemiology-colorectal-cancer-consortium-gecco.html). The derived data generated in this research will be shared on reasonable request to the corresponding author with permission of the Fred Hutchinson Cancer Center.

## Supplementary Material

pkab029_Supplementary_Data
