# Supplementary material for: Nongenetic Determinants of Risk for Early-Onset Colorectal Cancer
Source: JNCI Cancer Spectr. 2021 May 20;5(3):pkab029. doi: 10.1093/jncics/pkab029 (PMC8134523; doi:10.1093/jncics/pkab029)
Supplement: pkab029_Supplementary_Data [file pkab029_supplementary_data.pdf]

SUPPLEMENTARY METHODS

Harmonization of lifestyle and environmental data

Data harmonization was conducted at the coordinating center, the Fred Hutchinson Cancer Center in Seattle, WA, and performed using a standardized protocol. Demographic, lifestyle, dietary, and pharmacological information was ascertained by either self-report using in-person interviews and/or structured questionnaires, or via direct measurement. All self-reported variables were collected at the reference time for each study, defined as patient selection or blood collection for cohort studies, and 1-2 years prior to selection for case-control studies in order to ensure exposures were assessed before cancer diagnoses. For studies that captured height and BMI via direct measurement, variables were captured at the reference time of each respective study. The iterative, data harmonization procedure reconciled each respective studies' protocols and data collection methodology. Briefly, the first step involved defining common data elements (CDEs), which were utilized to map study-specific data elements identified from questionnaires and data dictionaries. CDEs were transformed and collated into a single dataset with common definitions, standardized values within a permissible range, and uniform coding. Outliers were truncated to the minimum or maximum permissible value based on the established range for each respective variable. Final data were reviewed for quality assurance.

Height

Height was defined in centimeters based on either self-report or via direct measurement. In our analyses, height was scaled to represent increments of 10 cm.

Body mass index

Body mass index (BMI) was estimated from self-report or via direct measurement of body weight (kg) divided by height (m^2). In our analyses, BMI was scaled to represent increments of 5 (i.e., BMI/5), and BMI levels <18.5 were set to missing.

Education

Highest level of education was categorized as follows: less than high school degree, high school degree or completed GED, some college or technical school, and college or graduate degree.

Diabetes and

Self-reported type 2 diabetes was categorized as a binary yes/no variable.

Sedentary lifestyle

Sedentary lifestyle was estimated by summing hours per week of moderate and/or vigorous physical activity, leisure-time, and undifferentiated activities. Self-reported leisure and occupational activity were self-explanatory, whereas undifferentiated activities included the following tasks: housework (e.g., cleaning, washing, cooking, child care), gardening, walking (e.g., walking to work, shopping, leisure), cycling (e.g., cycling to work and for leisure), and physical exercise (e.g., aerobics, swimming, jogging, tennis, line dancing). Participants were required to interpret if said activities were vigorous or moderate and retrospectively determine the number of hours per week. The variable was dichotomized as no if total sum >=1 hour/week, and yes if total sum <1 hour/week.

Pack-years of smoking

Pack-years of smoking were calculated by multiplying the average number of packs of cigarettes smoked per day by smoking duration in years. Smoking pack-years were harmonized across studies using sex- and study-specific quartiles, with quartile cutoffs based on the distribution within the sex-specific controls of each study. The variable was categorized as follows: 0 was assigned for never-smokers, 1 was assigned for ≤ first quartile, 2 was assigned for > first quartile but ≤ second quartile, 3 was assigned for > second

quartile but ≤ third quartile, and 4 was assigned for > third quartile. This variable was modelled as continuous variable throughout the analysis.

Alcohol consumption
Consumption of alcoholic beverages was estimated based on grams of alcohol per day (g/day) by summing the daily consumption of alcohol content per beverage. Participants were subsequently grouped as follows: non- and occasional drinkers (< 1 g/day), light-to-moderate drinkers (1-28 g/day), and heavy drinkers (>28 g/day).

Aspirin use
Aspirin use was defined as "yes" if a person regularly used aspirin in the reference time period, and "no" otherwise.

NSAID use
NSAID use was defined as "yes" if a person regularly used non-aspirin NSAIDs in the reference time period, and "no" otherwise.

Dietary variables
Dietary factors were assessed using food frequency questionnaires (FFQs) or diet history. Fruit, vegetable, and red or processed meat consumption were measured using servings per day. Whereas, fiber intake was represented in terms of grams per day, total folate intake as µg per day, and total calcium intake as mg per day. All dietary variables were recoded into sex- and study-specific quartiles, with quartile cutoffs based on the distribution within the sex-specific controls of each study, and modelled as continuous variables. Several studies had less variation in dietary intake, primarily as a result of fewer questions, with resulted in less than 4 intake categories for certain factors. In these instances, dietary intake was only assigned to the 2nd and 3rd quartiles. Total calcium intake (mg/day) was ascertained based on both the milligrams per day (mg/day) of calcium consumption in foods (i.e., dietary) and through dietary supplements (i.e., single, multivitamins, and antacids) when available. For studies that entered dietary supplement data as "regular user" versus "nonuser", we assumed regular use was 500 mg/day, 500 mg/single tablet, or 130 mg/multivitamin tablet (i.e., the generic dose in supplements). Folate and folic acid intake were determined based on micrograms per day (µg/day) of folate from foods (i.e., dietary folate) and µg/day of folic acid through dietary supplements (i.e., single or multivitamins) when available. To account for the higher bioavailability of synthetic folic acid compared with folate intake through food, we estimated total folate intake in terms of dietary folate equivalents (DFE): total µg DFE = µg of dietary folate + 1.7 x µg folic acid from supplements. Because enrollment periods for several studies overlapped or followed the period of folic acid fortification (1996-1998), these studies accounted for folic acid fortification when calculating total dietary folate intake by entering dietary folate intake as µg of dietary folate due to natural sources + 1.7 x µg folic acid from fortified foods. For studies that entered dietary supplement data as "regular user" versus "nonuser", we assumed regular use was 400 µg/day or 400 µg/multivitamin tablet (i.e., the generic dose in supplements). Total energy consumption was estimated in kcal/day, rescaled using its standard error, and modeled as a continuous variable.

SUPPLEMENTARY TABLES

Supplementary Table 1: Study descriptions

Study Name	Study Acronym	Design	Country	Reference Time[h]	Early-onset Cases	Early-onset Controls	Late-Onset Cases	Late-Onset Controls
Colon Cancer Family Registry	CCFR[a]	Case-control	Canada, Australia, and United States	1996-2009	2,033	993	2,763	2,102
Colorectal Cancer Genetics & Genomics, Spanish Study	CRCGEN	Case-control	Spain	1996-2015	54	97	831	925
Darmkrebs: Chancen der Verhütung durch Screening	DACHS[b,c]	Case-control	Germany	2003-2015	166	118	3,352	2,670
Diet, Activity and Lifestyle Study	DALS[d]	Case-control	United States	1990-1993	83	89	1,016	1,074
European Prospective Investigation into Cancer and Nutrition	EPIC	Cohort	France, Germany, Greece, Italy, Netherlands, Spain, Sweden, and United Kingdom	1992-1999	90	28	1,932	2,289
Kentucky Case-Control Study	Kentucky[e]	Case-control	United States	2001-2010	102	76	859	1,056
Leeds Colorectal Cancer Study	LCCS	Case-control	United Kingdom	1997-2011	85	25	1,324	658
Molecular Epidemiology of Colorectal Cancer	MECC[f]	Case-control	Israel	1972-2015	378	213	4,808	4,116
North Carolina Colon Cancer Studies I	NCCCS I	Case-control	United States	1997-2000	21	30	221	434
North Carolina Colon Cancer Studies II	NCCCS II	Case-control	United States	2002-2010	86	60	509	626
Newfoundland Case-Control Study	NFCCR	Case-control	Canada	2000-2004	84	69	541	402
Nurses' Health Study	NHS[g]	Cohort	United States	1978-2013	33	49	810	1,206
UK Biobank	UKB	Cohort	United Kingdom	2006-2010	552	2,202	4,471	17,753

[a]Newcomb PA, et al. Colon Cancer Family Registry: an international resource for studies of the genetic epidemiology of colon cancer. Cancer epidemiology, biomarkers & prevention. 2007;16(11):2331-2343.

[b]Brenner H, at al. Protection from colorectal cancer after colonoscopy: a population-based, case-control study. Annals of internal medicine. 2011;154(1):22-30.

[c]Lilla C, et al. Effect of NAT1 and NAT2 genetic polymorphisms on colorectal cancer risk associated with exposure to tobacco smoke and meat consumption. Cancer epidemiology, biomarkers & prevention. 2006;15(1):99-107.

[d]Slattery ML, et al. Energy balance and colon cancer--beyond physical activity. Cancer research. 1997;57(1):75-80.

[e]Nock NL, et al. Associations between obesity and changes in adult BMI over time and colon cancer risk. Obesity (Silver Spring, Md). 2008;16(5):1099-1104.

[f]Poynter JN, et al. Statins and the risk of colorectal cancer. The New England journal of medicine. 2005;352(21):2184-2192.

[g]Belanger CF, et al. The nurses' health study. The American journal of nursing. 1978;78(6):1039-1040.

[h]Reference time for each study was defined as patient selection or blood collection for cohort studies, and 1-2 years prior to selection for case-control studies. Only the years for included participants were reported.

Supplementary Table 2: Baseline participant characteristics of participants aged ≥50 years[a]

Characteristic	Cases	Controls
N	23,437	35,311
Age, Mean (SD)	65.84 (9.31)	63.79 (8.27)
Sex, N (%)		
Female	10915 (46.6)	16285 (46.1)
Male	12522 (53.4)	19026 (53.9)
Disease Site, N (%)		
Proximal Colon	7413 (33.9)	--
Distal Colon	7321 (33.5)	
Rectum	7128 (32.6)	--
Education, highest level completed, N (%)		
< High school graduate	6790 (31.5)	7820 (25.5)
High school graduate or completed GED	4725 (21.9)	4523 (14.8)
Some college or technical school	4780 (22.2)	8509 (27.8)
≥ College graduate	5266 (24.4)	9766 (31.9)
Family History, N (%)		
No	14537 (81.0)	18663 (82.6)
Yes	3418 (19.0)	3928 (17.4)
Height, per 10 cm, Mean (SD)	16.85 (0.94)	16.87 (0.94)
BMI, per 5 kg/m^2, Mean (SD)	5.52 (0.94)	5.45 (0.91)
Red meat (servings/day), N (%)		
Quartile 1[b]	5430 (24.2)	9468 (27.2)
Quartile 2[b]	6042 (27.0)	10276 (29.6)
Quartile 3[b]	6052 (27.0)	9742 (28.0)
Quartile 4[b]	4876 (21.8)	5260 (15.1)
Processed meat (servings/day), N (%)		
Quartile 1[b]	3054 (15.1)	5599 (16.9)
Quartile 2[b]	7258 (35.8)	12572 (38.0)
Quartile 3[b]	7394 (36.4)	12141 (36.7)
Quartile 4[b]	2585 (12.7)	2753 (8.3)
Fruit (servings/day), N (%)		
Quartile 1[b]	6066 (27.3)	8823 (25.6)
Quartile 2[b]	7535 (33.9)	10095 (29.3)
Quartile 3[b]	4695 (21.1)	8026 (23.3)
Quartile 4[b]	3952 (17.8)	7482 (21.7)
Vegetable (servings/day), N (%)		
Quartile 1[b]	5104 (22.8)	8923 (25.9)
Quartile 2[b]	8034 (36.0)	9625 (27.9)
Quartile 3[b]	5295 (23.7)	8627 (25.0)
Quartile 4[b]	3905 (17.5)	7316 (21.2)

Total fiber (g/day)		
Quartile 1[b]	3687 (28.0)	3347 (24.8)
Quartile 2[b]	3399 (25.8)	3349 (24.8)
Quartile 3[b]	3075 (23.4)	3403 (25.2)
Quartile 4[b]	2998 (22.8)	3417 (25.3)
Total calcium intake (mg/day), N (%)		
Quartile 1[b]	4253 (19.0)	3765 (10.9)
Quartile 2[b]	9154 (40.8)	17924 (51.7)
Quartile 3[b]	5580 (24.9)	9159 (26.4)
Quartile 4[b]	3434 (15.3)	3834 (11.1)
Total folate intake (mcg/day), N (%)		
Quartile 1[b]	3846 (20.4)	3325 (10.4)
Quartile 2[b]	7169 (38.1)	17670 (55.3)
Quartile 3[b]	4785 (25.4)	7501 (23.5)
Quartile 4[b]	3022 (16.1)	3442 (10.8)
Sedentary lifestyle, N (%)		
No	7946 (77.4)	15994 (79.1)
Yes	2325 (22.6)	4218 (20.9)
Pack-years of smoking, N (%)		
Never smoker	9419 (50.7)	15800 (55.7)
Quartile 1[b]	1914 (10.3)	3003 (10.6)
Quartile 2[b]	2137 (11.5)	2964 (10.4)
Quartile 3[b]	2375 (12.8)	3097 (10.9)
Quartile 4[b]	2717 (14.6)	3502 (12.3)
Alcohol use (g/day), N (%)		
0 g/day	9686 (43.6)	11161 (32.3)
1-28 g/day	9480 (42.7)	18427 (53.4)
>28 g/day	3033 (13.7)	4950 (14.3)
Aspirin use, N (%)		
No	15519 (74.2)	23894 (73.3)
Yes	5402 (25.8)	8718 (26.7)
NSAID use, N (%)		
No	19056 (91.2)	28554 (87.6)
Yes	1833 (8.8)	4024 (12.4)
History of diabetes, N (%)		
No	18548 (84.8)	30588 (90.0)
Yes	3324 (15.2)	3398 (10.0)

[a]Age defined as the age of diagnosis of the first primary CRC for cases, and as the age at selection for controls. Abbreviations: BMI, body mass index; GED, general educational development; SD, standard deviation.
[b]Study and sex-specific quartiles.

Supplementary Table 3: Missing proportions across the complete consortia dataset that was used for mean imputation

Lifestyle and Environmental Risk Factor	Cases	Controls
N	67,168	710,377
Height (% missing)	7.6%	1.8%
BMI (% missing)	4.2%	1.9%
Red meat (servings/day) (% missing)	6.5%	5.2%
Processed meat (servings/day) (% missing)	16.3%	6.3%
Fruit (servings/day) (% missing)	7.4%	6.2%
Vegetable (servings/day) (% missing)	7.0%	6.2%
Total fiber (g/day) (% missing)	19.9%	19.2%
Total calcium intake (mg/day) (% missing)	8.9%	6.0%
Total folate intake (mcg/day) (% missing)	12.2%	6.4%
Sedentary lifestyle (% missing)	27.8%	23.6%
Pack-years of smoking (% missing)	12.3%	13.7%
Alcohol use (g/day) (% missing)	8.9%	6.2%
Aspirin use (% missing)	5.2%	3.0%
NSAIDS use (% missing)	4.9%	3.0%
History of diabetes (% missing)	5.0%	1.9%

Abbreviations: BMI, body mass index; NSAID, nonsteroidal anti-inflammatory drug.

Supplementary Table 4: Risk estimates for late-onset CRC associated with anthropometric, dietary, lifestyle, and pharmacological risk factors

Lifestyle and Environmental Risk Factor[d]	Minimally-adjusted Models[a]		Multivariable Model[b]	
	OR (95% CI)	P-value	OR (95% CI)	P-value
Anthropometric				
BMI (per 5 kg/m^2)	1.14 (1.11, 1.16)	<0.001	1.12 (1.09, 1.14)	<0.001
Height (per 10 cm)	1.02 (0.99, 1.05)	0.19	1.04 (1.01, 1.07)	0.01
Lifestyle				
Pack-years of smoking	1.07 (1.05, 1.08)	<0.001	1.05 (1.03, 1.07)	<0.001
Sedentary lifestyle	1.13 (1.04, 1.23)	0.003	1.11 (1.02, 1.22)	0.02
Alcohol use (0 g/d)	1.12 (1.17, 1.28)	<0.001	1.20 (1.14, 1.26)	<0.001
Alcohol use (>28 g/d)	1.31 (1.23, 1.40)	<0.001	1.23 (1.15, 1.32)	<0.001
Lower educational attainment, highest level completed	1.10 (1.08, 1.12)	<0.001	1.06 (1.04, 1.08)	<0.001
History of diabetes	1.25 (1.18, 1.32)	<0.001	1.20 (1.12, 1.28)	<0.001
Dietary				
Lower total folate intake (mcg/day)[c]	1.16 (1.13, 1.19)	<0.001	1.04 (1.01, 1.07)	0.009
Lower fruit intake (servings/day)[c]	1.13 (1.11, 1.15)	<0.001	1.06 (1.04, 1.08)	<0.001
Lower vegetable intake (servings/day)[c]	1.07 (1.05, 1.09)	<0.001	1.01 (0.99, 1.04)	0.29
Greater red meat intake (servings/day)[c]	1.13 (1.11, 1.15)	<0.001	1.07 (1.05, 1.10)	<0.001
Greater processed meat intake (servings/day)[c]	1.14 (1.11, 1.17)	<0.001	1.06 (1.03, 1.09)	<0.001
Lower total fiber intake (g/day)[c]	1.18 (1.15, 1.21)	<0.001	1.10 (1.06, 1.14)	<0.001
Lower total calcium intake (mg/day)[c]	1.20 (1.17, 1.23)	<0.001	1.13 (1.10, 1.16)	<0.001
Pharmacological				
No aspirin use	1.30 (1.25, 1.37)	<0.001	1.41 (1.34, 1.48)	<0.001
No NSAID use	1.36 (1.27, 1.46)	<0.001	1.40 (1.30, 1.51)	<0.001

[a]Logistic regression models include individual non-genetic factors, and were adjusted for age, sex, study, family history, history of screening, and total energy consumption (for dietary factors). Abbreviations: BMI, body mass index; CI, confidence interval; NSAID, nonsteroidal anti-inflammatory drug; OR, odds ratio.

[b]Logistic regression model includes all non-genetic factors, and was adjusted for age, sex, study, family history, history of screening, and total energy consumption.

[c]Dietary variables were harmonized across studies by sex- and study-specific quartiles, and assigned values 0,1,2,3 in the order of increasing risk. These variables were treated as continuous variables in the analysis.

[d]The referent category for each categorical factor was defined as the following: presence of a sedentary lifestyle (no), alcohol intake (1-28 g/day), educational attainment (≥ college graduate), history of diabetes (no), aspirin use (yes), and NSAID use (yes).

Supplementary Table 5: Association of NSAID use with early-onset CRC, after exclusion of individuals with IBD and those missing IBD diagnostic information

	Minimally-adjusted Model[a]		Multivariable Model[b]	
	OR (95% CI)	P-value	OR (95% CI)	P-value
No NSAID use[c]	1.40 (1.14, 1.72)	0.002	1.42 (1.15, 1.76)	0.001

[a]Logistic regression model includes NSAID use, age, sex, study, family history, and history of screening. Abbreviations: CI, confidence interval; NSAID, nonsteroidal anti-inflammatory drug; OR, odds ratio.

[b]Logistic regression model includes all non-genetic factors, and was adjusted for age, sex, study, family history, history of screening, and total energy consumption.

[c]The referent category for each categorical factor was defined as the following: presence of a sedentary lifestyle (no), alcohol intake (1-28 g/day), educational attainment (≥ college graduate), history of diabetes (no), aspirin use (yes), and NSAID use (yes).

Supplementary Table 6: Association between anthropometric, dietary, lifestyle, and pharmacological risk factors and early-onset CRC risk, stratified by granular anatomic subsite (i.e., proximal colon, distal colon, and rectum)

Lifestyle and Environmental Risk Factor[d]	Proximal Colon Cancer[a]		Distal Colon Cancer[a]		Rectal Cancer[a]		Proximal vs. Distal Colon[b]	Distal Colon vs. Rectum[b]	Proximal Colon vs. Rectum[b]
	OR (95% CI)	P-value	OR (95% CI)	P-value	OR (95% CI)	P-value	P-value	P-value	P-value
Anthropometric									
BMI (per 5 kg/m^2)	1.04 (0.97, 1.12)	0.23	1.04 (0.97, 1.11)	0.25	0.99 (0.93, 1.06)	0.84	0.93	0.22	0.21
Height (per 10 cm)	0.98 (0.88, 1.10)	0.74	1.05 (0.95, 1.17)	0.32	1.03 (0.93, 1.13)	0.57	0.27	0.70	0.43
Lifestyle									
Pack-years of smoking	0.97 (0.91, 1.04)	0.40	1.00 (0.94, 1.06)	0.99	0.99 (0.94, 1.05)	0.73	0.45	0.72	0.66
Sedentary lifestyle	1.05 (0.70, 1.56)	0.83	1.08 (0.75, 1.57)	0.68	1.09 (0.78, 1.53)	0.63	0.89	0.97	0.86
Alcohol use (0 g/d)	1.33 (1.12, 1.59)	0.001	1.26 (1.07, 1.49)	0.006	1.30 (1.11, 1.53)	0.001	0.60	0.77	0.80
Alcohol use (>28 g/d)	1.18 (0.91, 1.53)	0.21	1.41 (1.10, 1.80)	0.007	1.34 (1.08, 1.67)	0.009	0.26	0.69	0.41
Lower educational attainment	1.16 (1.07, 1.25)	<0.001	1.12 (1.04, 1.20)	0.003	1.13 (1.06, 1.21)	<0.001	0.45	0.67	0.70
History of diabetes	1.16 (0.78, 1.73)	0.45	1.25 (0.87, 1.80)	0.24	1.28 (0.90, 1.81)	0.16	0.75	0.93	0.68
Dietary									
Lower total folate intake (mcg/day)[c]	1.13 (1.01, 1.27)	0.03	1.19 (1.07, 1.33)	0.001	1.24 (1.11, 1.37)	<0.001	0.41	0.61	0.19
Lower fruit intake (servings/day)[c]	1.05 (0.98, 1.13)	0.13	1.02 (0.95, 1.09)	0.58	1.10 (1.03, 1.17)	0.004	0.40	0.06	0.34
Lower vegetable intake (servings/day)[c]	1.07 (0.99, 1.16)	0.08	0.98 (0.91, 1.06)	0.66	1.08 (1.01, 1.16)	0.03	0.06	0.04	0.95
Greater red meat intake (servings/day)[c]	1.15 (1.07, 1.23)	<0.001	1.09 (1.01, 1.16)	0.02	1.12 (1.05, 1.19)	0.001	0.19	0.45	0.53
Greater processed meat intake (servings/day)[c]	1.05 (0.93, 1.18)	0.47	1.10 (0.98, 1.22)	0.11	1.09 (0.98, 1.21)	0.11	0.51	0.97	0.52
Lower total fiber intake (g/day)[c]	1.24 (1.08, 1.43)	0.002	1.06 (0.94, 1.21)	0.33	1.30 (1.14, 1.48)	<0.001	0.05	0.006	0.57
Lower total calcium intake (mg/day)[c]	1.11 (0.98, 1.26)	0.11	1.18 (1.06, 1.33)	0.003	1.24 (1.11, 1.39)	<0.001	0.37	0.42	0.10
Pharmacological									
No aspirin use	1.08 (0.81, 1.43)	0.61	1.18 (0.90, 1.55)	0.23	1.04 (0.81, 1.34)	0.75	0.59	0.39	0.79
No NSAID use	1.29 (1.02, 1.64)	0.04	1.44 (1.14, 1.82)	0.002	1.66 (1.31, 2.09)	<0.001	0.47	0.30	0.09

[a]Multinomial logistic regression models include individual non-genetic factors, and were adjusted for age, sex, study, family history, and total energy consumption (for dietary factors). Abbreviations: BMI, body mass index; CI, confidence interval; NSAID, nonsteroidal anti-inflammatory drug; OR, odds ratio.

[b]Chi-square test for contrasts in multinomial models

[c]Dietary variables were harmonized across studies by sex- and study-specific quartiles, and assigned values 0,1,2,3 in the order of increasing risk. These variables were treated as continuous variables in the analysis.

[d]The referent category for each categorical factor was defined as the following: presence of a sedentary lifestyle (no), alcohol intake (1-28 g/day), educational attainment (≥ college graduate), history of diabetes (no), aspirin use (yes), and NSAID use (yes).

Supplementary Table 7: Sensitivity analyses of minimally-adjusted relative risks for early-onset CRC associated with anthropometric, dietary, lifestyle, and pharmacological risk factors

Lifestyle and Environmental Risk Factor[c]	Multiple Imputation Analysis[a]		Complete-Case Analysis[a]			
	OR (95% CI)	P-value	Cases, N	Controls, N	OR (95% CI)	P-value
Anthropometric						
BMI (per 5 kg/m^2)	1.06 (1.01, 1.12)	0.01	3,148	2,906	1.07 (1.01, 1.13)	0.01
Height (per 10 cm)	1.05 (0.98, 1.13)	0.18	3,253	2,944	1.07 (0.98, 1.15)	0.12
Lifestyle						
Pack-years of smoking	1.00 (0.95, 1.04)	0.93	3,084	2,680	1.00 (0.96, 1.05)	0.92
Sedentary lifestyle	1.13 (0.88, 1.47)	0.34	646	1,310	1.02 (0.74, 1.40)	0.92
Alcohol use (0 g/d)	1.25 (1.11, 1.40)	<0.001	2,987	2,886	1.24 (1.09, 1.41)	<0.001
Alcohol use (>28 g/d)	1.34 (1.13, 1.58)	<0.001	2,987	2,886	1.30 (1.08, 1.57)	0.007
Lower educational attainment, highest level completed	1.12 (1.06, 1.18)	<0.001	3,203	2,886	1.16 (1.10, 1.23)	<0.001
History of diabetes	1.21 (0.92, 1.60)	0.18	3,250	2,865	1.25 (0.93, 1.69)	0.14
Dietary						
Lower total folate intake (mcg/day)[b]	1.16 (1.05, 1.29)	0.003	2,159	2,145	1.16 (1.06, 1.26)	0.001
Lower fruit intake (servings/day)[b]	1.07 (1.02, 1.12)	0.007	3,170	2,887	1.06 (1.01, 1.12)	0.02
Lower vegetable intake (servings/day)[b]	1.05 (1.00, 1.10)	0.07	3,204	2,898	1.07 (1.01, 1.13)	0.03
Greater red meat intake (servings/day)[b]	1.12 (1.06, 1.18)	<0.001	3,159	2,882	1.12 (1.06, 1.17)	<0.001
Greater processed meat intake (servings/day)[b]	1.14 (1.04, 1.26)	0.007	1,675	2,025	1.13 (1.04, 1.24)	0.005
Lower total fiber intake (g/day)[b]	1.17 (1.07, 1.27)	<0.001	1,267	826	1.15 (1.04, 1.27)	0.007
Lower total calcium intake (mg/day)[b]	1.19 (1.09, 1.29)	<0.001	2,829	2,673	1.15 (1.05, 1.26)	0.002
Pharmacological						
No aspirin use	1.08 (0.90, 1.31)	0.41	3,303	2,933	1.12 (0.91, 1.38)	0.28
No NSAID use	1.44 (1.22, 1.69)	<0.001	3,280	2,922	1.45 (1.22, 1.72)	<0.001

[a]Logistic regression models were adjusted for age, sex, study, family history, and total energy consumption (for dietary factors).
Abbreviations: BMI, body mass index; CI, confidence interval; NSAID, nonsteroidal anti-inflammatory drug; OR, odds ratio.
[b]Dietary variables were harmonized across studies by sex- and study-specific quartiles, and assigned values 0,1,2,3 in the order of increasing risk. These variables were treated as continuous variables in the analysis.
[c]The referent category for each categorical factor was defined as the following: presence of a sedentary lifestyle (no), alcohol intake (1-28 g/day), educational attainment (≥ college graduate), history of diabetes (no), aspirin use (yes), and NSAID use (yes).

Supplementary Table 8: Sensitivity analysis of minimally-adjusted relative risks with complete case data for early-onset CRC associated with anthropometric, dietary, lifestyle, and pharmacological risk factors, stratified by anatomic subsite

Lifestyle and Environmental Risk Factor[d]	Colon Cancer[a]		Rectal Cancer[a]		Colon vs. Rectum[b]
	OR (95% CI)	P-value	OR (95% CI)	P-value	P-value
Anthropometric					
BMI (per 5 kg/m^2)	1.08 (1.02, 1.14)	0.005	1.03 (0.97, 1.10)	0.36	0.16
Height (per 10 cm)	1.03 (0.95, 1.12)	0.50	1.03 (0.94, 1.14)	0.53	0.97
Lifestyle					
Pack-years of smoking	0.99 (0.95, 1.04)	0.79	0.99 (0.94, 1.05)	0.84	0.98
Sedentary lifestyle	1.16 (0.87, 1.56)	0.32	1.09 (0.76, 1.56)	0.64	0.75
Alcohol use (0 g/d)	1.26 (1.10, 1.44)	<0.001	1.28 (1.09, 1.50)	0.003	0.84
Alcohol use (>28 g/d)	1.30 (1.07, 1.58)	0.008	1.35 (1.09, 1.69)	0.007	0.73
Lower educational attainment, highest level completed	1.12 (1.05, 1.18)	<0.001	1.13 (1.06, 1.21)	<0.001	0.68
History of diabetes	1.17 (0.86, 1.59)	0.32	1.26 (0.89, 1.78)	0.19	0.66
Dietary					
Lower total folate intake (mcg/day)[c]	1.16 (1.06, 1.27)	0.002	1.25 (1.12, 1.39)	<0.001	0.16
Lower fruit intake (servings/day)[c]	1.05 (0.99, 1.10)	0.10	1.09 (1.03, 1.16)	0.004	0.16
Lower vegetable intake (servings/day)[c]	1.03 (0.97, 1.09)	0.32	1.08 (1.01, 1.16)	0.04	0.23
Greater red meat intake (servings/day)[c]	1.12 (1.06, 1.18)	<0.001	1.12 (1.05, 1.19)	<0.001	0.99
Greater processed meat intake (servings/day)[c]	1.06 (0.97, 1.16)	0.21	1.09 (0.98, 1.21)	0.12	0.62
Lower total fiber intake (g/day)[c]	1.12 (1.01, 1.25)	0.03	1.28 (1.12, 1.46)	<0.001	0.06
Lower total calcium intake (mg/day)[c]	1.16 (1.05, 1.27)	0.002	1.26 (1.13, 1.41)	<0.001	0.14
Pharmacological					
No aspirin use	1.15 (0.93, 1.43)	0.20	1.04 (0.80, 1.34)	0.78	0.43
No NSAID use	1.34 (1.12, 1.60)	0.002	1.65 (1.31, 2.08)	<0.001	0.09

[a]Multinomial logistic regression models include individual non-genetic factors, and were adjusted for age, sex, study, family history, and total energy consumption (for dietary factors). Abbreviations: BMI, body mass index; CI, confidence interval; NSAID, nonsteroidal anti-inflammatory drug; OR, odds ratio.

[b]Chi-square test for contrasts in multinomial models

[c]Dietary variables were harmonized across studies by sex- and study-specific quartiles, and assigned values 0,1,2,3 in the order of increasing risk. These variables were treated as continuous variables in the analysis.

[d]The referent category for each categorical factor was defined as the following: presence of a sedentary lifestyle (no), alcohol intake (1-28 g/day), educational attainment (≥ college graduate), history of diabetes (no), aspirin use (yes), and NSAID use (yes).
